# Evidence-Based Framework to Manage Cyanobacteria and Cyanotoxins in Water and Sludge from Drinking Water Treatment Plants

**DOI:** 10.3390/toxins14060410

**Published:** 2022-06-15

**Authors:** Farhad Jalili, Saber Moradinejad, Arash Zamyadi, Sarah Dorner, Sébastien Sauvé, Michèle Prévost

**Affiliations:** 1 Department of Civil, Mineral and Mining Engineering, Polytechnique Montréal, Montréal, QC H3C 3A7, Canada; farhad.jalili@polymtl.ca (F.J.); sarah.dorner@polymtl.ca (S.D.); michele.prevost@polymtl.ca (M.P.); 2 Faculty of Engineering and Information Technology, University of Melbourne, Melbourne, VIC 3010, Australia; arash.zamyadi@unimelb.edu.au; 3 Department of Chemistry, University of Montréal, Montréal, QC H3C 3J7, Canada; sebastien.sauve@umontreal.ca

**Keywords:** cyanobacteria, cyanotoxins, pre-oxidation, sludge, accumulation, management, water treatment plant

## Abstract

Freshwater bodies and, consequently, drinking water treatment plants (DWTPs) sources are increasingly facing toxic cyanobacterial blooms. Even though conventional treatment processes including coagulation, flocculation, sedimentation, and filtration can control cyanobacteria and cell-bound cyanotoxins, these processes may encounter challenges such as inefficient removal of dissolved metabolites and cyanobacterial cell breakthrough. Furthermore, conventional treatment processes may lead to the accumulation of cyanobacteria cells and cyanotoxins in sludge. Pre-oxidation can enhance coagulation efficiency as it provides the first barrier against cyanobacteria and cyanotoxins and it decreases cell accumulation in DWTP sludge. This critical review aims to: (i) evaluate the state of the science of cyanobacteria and cyanotoxin management throughout DWTPs, as well as their associated sludge, and (ii) develop a decision framework to manage cyanobacteria and cyanotoxins in DWTPs and sludge. The review identified that lab-cultured-based pre-oxidation studies may not represent the real bloom pre-oxidation efficacy. Moreover, the application of a common exposure unit CT (residual concentration × contact time) provides a proper understanding of cyanobacteria pre-oxidation efficiency. Recently, reported challenges on cyanobacterial survival and growth in sludge alongside the cell lysis and cyanotoxin release raised health and technical concerns with regards to sludge storage and sludge supernatant recycling to the head of DWTPs. According to the review, oxidation has not been identified as a feasible option to handle cyanobacterial-laden sludge due to low cell and cyanotoxin removal efficacy. Based on the reviewed literature, a decision framework is proposed to manage cyanobacteria and cyanotoxins and their associated sludge in DWTPs.

## 1. Introduction

A cyanobacterial bloom occurrence may result in metabolite (cyanotoxins and taste and odor agents) production and release, which is considered a widespread problem in drinking water resources around the world [1,2,3,4,5,6,7,8,9,10].

Conventional treatment processes, including coagulation, flocculation, sedimentation, and filtration, are widely applied to remove cyanobacterial cells and cell-bound cyanotoxins [11,12,13,14]. However, conventional treatment processes may not be able to remove dissolved metabolites (e.g., cyanotoxins) efficiently [12,15,16,17,18,19,20,21,22]. Moreover, toxic cyanobacterial breakthrough has been reported in the effluent of conventional treatment processes and even after post-oxidation [18]. Therefore, additional treatment such as oxidation or powdered (PAC)/granular activated carbon (GAC) may be required to control dissolved metabolites [23,24,25,26].

Pre-oxidation enhances cyanobacteria cell removal during the coagulation/sedimentation process [27,28,29,30,31,32,33,34] and may decrease the cell breakthrough potential from the downflow processes. However, it is reported that pre-oxidation may cause cyanobacteria cell damage (decrease in cell viability) and cell-bound cyanotoxin release [35,36,37]. The level of cell lysis/damage and cyanotoxin degradation/release following pre-oxidation depends on the oxidation exposure (CT as residual concentration x contact time), and it is the driver to find the best pre-oxidation practice against cyanobacteria and cyanotoxins [38,39].

Furthermore, conventional treatment processes cause cell accumulation in drinking water treatment plants’ (DWTPs) sludge, even in DWTPs with low cyanobacterial cell numbers in the intake water [13,18,40,41,42,43,44]. Several studies have demonstrated that cyanobacterial cells could survive in the stored sludge and release cyanotoxins for up to 12 days [13,40,45,46,47,48,49,50,51,52]. Recent studies revealed a new challenge on the probability of extended survival time and even cyanobacterial growth during sludge storage [53,54,55]. Thus, recycling the supernatant of stored cyanobacteria-laden sludge to the head of the DWTPs can increase health-related concerns [55,56]. Such challenges highlight the importance of the treatment and management of cyanobacteria-laden sludge [57,58,59].

The objectives of this study are to: (1) critically review shreds of evidence of the pre-oxidation impact on the cultured-based and natural bloom studies, (2) perform a critical review of the fate of cyanobacteria and cyanotoxins in conventional treatment plants’ sludge and during sludge storage, and (3) develop an operational decision framework to determine the best practice to minimize risks associated with cyanobacteria and cyanotoxin presence in DWTPs.

This critical review provides insight into the fate of cyanobacteria and their associated metabolites throughout DWTPs and their sludge; furthermore, a practical decision framework to mitigate health and operational risks is developed.

## 2. Impact of Conventional Treatment on Cyanobacteria and Cyanotoxin Accumulation in Sludge

Different studies have reported that conventional treatment processes can remove 62–99% of the cyanobacterial cells in DWTPs [13,18,40,41,42,56]. It has been demonstrated that potential toxic cyanobacterial cells such as *Microcystis*, *Dolichospermum*, and *Aphanocapsa* can be removed using conventional processes [12,56,60,61]. 

The long-term monitoring of a high-risk DWTP (Lake Champlain—Quebec) during cyanobacterial bloom seasons from 2008 to 2011 showed an extreme accumulation of cyanobacteria cells (up to 10^7^ cells/mL) and cyanotoxins (up to 60 μg/L microcystin-LR (MC-LR)) in the sludge of the clarifier [12,18]. Monitoring of the same DWTP in 2017 showed that cyanobacteria cell accumulation in the sludge holding tank was up to 31-fold higher than taxonomic cell counts in the intake water [56]. An investigation of four DWTPs in the Great Lakes (Ontario) with low cyanobacterial cell influx (<1000 cells/mL) revealed that cyanobacterial cells and cyanotoxins accumulated in the sludge by up to 100 and 12 times higher than the raw water, respectively [44]. Zamyadi, et al. [57] reported a 406% and 2600% cell count increase in the thickened and centrifuged sludge, respectively, in a DWTP equipped with dissolved air flotation (DAF). A similar high accumulation was also reported for the backwash of the direct filtration process [45].

**Figure 1:**
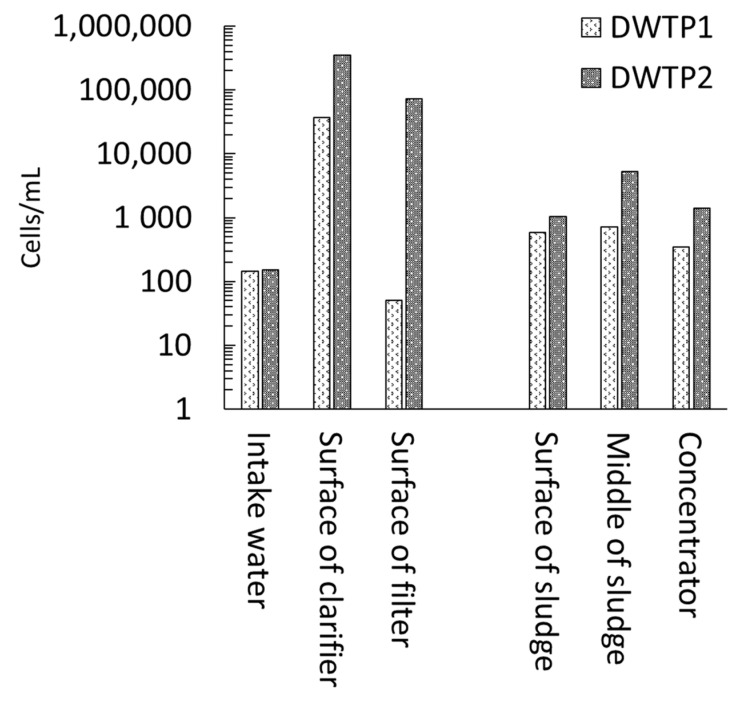
Cyanobacterial accumulation in two low risk DWTPs (maximum influx cell: <500 cells/mL). Only DWTP1 had pre-ozonation. The average values are from August to October 2011, adapted from [43].

Pre-oxidation may decrease the risk of cyanobacterial accumulation in the clarifier and sludge [43]. Two DWTPs (the same source for intake water with low cyanobacteria cells; maximum < 500 cells/mL) with chemically enhanced conventional treatment processes were studied, but only one of the DWTPs was equipped by pre-ozonation [43]. Pre-ozonation (initial concentration: 0.3–0.8 mg/L, contact time: 6.3 min) decreased cell accumulation in the surface of the clarifier and filters by up to 1450 times as compared to the DWTP without pre-ozonation. Accordingly, an up to 7 times lower cell accumulation was observed in the sludge of the DWTP with pre-ozonation (Figure 1).

In many DWTPs, the supernatant of the stored sludge is recycled to the head of the plant as the spent filter backwash water [45,57,62]. A full-scale study on a low-risk DWTP (3400 cells/mL at the intake water) documented that cyanobacterial cell counts in intake water increased by up to 43% after recycling the supernatant. Surprisingly, 80% of the transferred cells from the supernatant water were viable [60]. A recent laboratory investigation on intake water that contained 1 × 10^6^ cells/mL of cultured *M. aeruginosa* reported that although conventional treatment maintained the treated effluent parameters at below WHO and USEPA guidelines, recycling of the sludge supernatant resulted in an additional increase in cells and cyanotoxins levels in the influent by up to 7 × 10^4^ cells/mL and 0.26 µg/L MC-LR, respectively [63].

## 3. Pre-Oxidation Impact on Cyanobacteria Cells, Viability, and Cyanotoxins

### 3.1. Impact of Pre-Oxidation on Cyanobacteria Cell Counts

Cyanobacterial entry into DWTPs can be dampened by using pre-oxidation. Pre-oxidation may cause cell lysis, damage, and cyanotoxin release and degradation. Several studies were conducted to evaluate the pre-oxidation impact on cyanobacteria (e.g., cell viability and lysis) and cyanotoxins (release and degradation). A recent study tried to map the treatment barriers against cyanobacteria cells and cyanotoxins in drinking water facilities [10]. The results showed that the efficiency of the multi-barrier approach depends on the species present, metabolite concentration, and pre-oxidation dose [10]. Table 1, Table 2, Table 3 and Table 4 summarize the literature on the impact of pre-oxidation on cyanobacteria (cultured-based and natural blooms) for four common oxidants (chlorine, ozone, potassium permanganate, and hydrogen peroxide). Table 1, Table 2, Table 3 and Table 4 show that CT (residual concentration × contact time) is a main driver of cyanobacterial cell lysis, damage, cyanotoxin release, and degradation.

**Figure 2:**
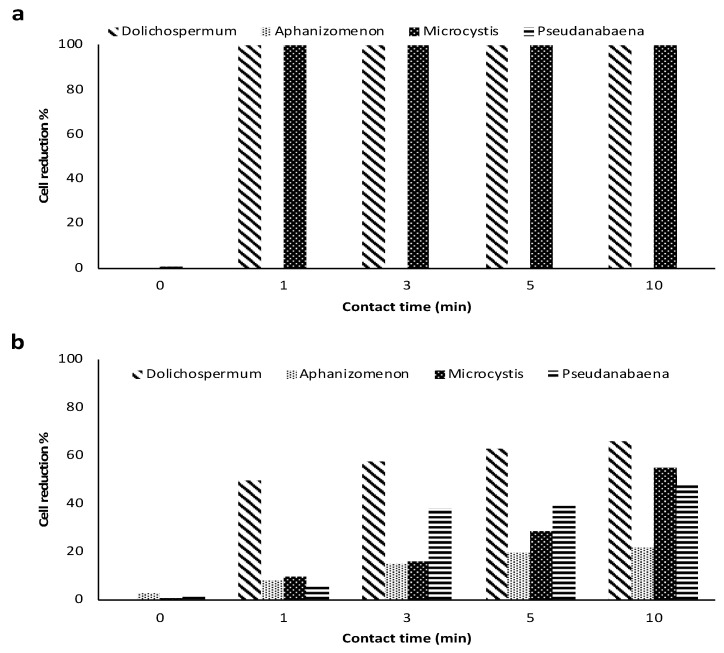
Comparison of cell count reduction following ozonation (2 mg/L) in the (**a**) cultured *Dolichospermum*, *Microcystis* [64], (**b**) Natural bloom [65].

Although some studies have reported a reduction of more than 90% in taxonomic cell counts following pre-oxidation in the lab-cultured cells, Zamyadi, et al. [35] reported a cell reduction of 70% at high chlorine exposure (CT 296 mg min/L). Fan, et al. [37] showed a limited impact of chlorine exposure (CT 104 mg min/L) on the taxonomic cell counts of *Microcystis aeruginosa* (logarithmic phase). These observations might be related to the cyanobacteria stage of life and agglomeration. Furthermore, comparing the taxonomic cell count percentage in cultured-based and natural bloom studies demonstrates the lower impact of pre-oxidation during natural blooms. Figure 2 exhibits a lower impact of pre-ozonation (2 mg/L) on cell number reduction in a natural bloom in comparison with lab-cultured cyanobacteria.

Table 1, Table 2, Table 3 and Table 4 and Figure 2 show that pre-oxidation, even at high CTs, may not be able to cause complete cell lysis. Consequently, it is important to clarify how far the pre-oxidation can cause viability loss and cyanotoxin release.

**Table 1:** Summary of the literature on the impact of pre-chlorination on cyanobacteria (cultured and natural blooms). HV: high viability, LV: low viability, DV: development stage, MA: maintenance stage.

Dominant Cyanobacteria (Cell Density)	Lab/Field	Cl_2_ Dose (mg/L)	Contact Time (min)	CT (mg min/L)	Cell Count Reduction %	Cell Viability %	Toxins	Reference	Comment
*Microcystis* (2 × 10^6^ cells/mL)	Lab	1–2	-	min. 15 max. 90	-	min. 83 max. 18.4	99% degradation	[66]	Saline solution; exact dose and contact time were not provided; no residual; CT evaluation weak; no cell-bound
*D. circinalis* (46,000 cells/mL)	Lab	2 3	0–60	min. 1.8 max. 50	-	min. 15% 0 for CT 5.8	>100% release (CT 5.8) >90 degradation (CT 50)	[67]	River water; using fluorescein diacetate (FDA) for viability
*Microcystis* (6 × 10^4^ cells/mL (2.5 × 10^5^ cells/mL) (5 × 10^5^ cells/mL)	Lab	2 4.5 10	0–60	min. 3 max. 296.1	max. 76%	-	>100% release (CT 5) >90 degradation (CT 35)	[35]	River water, ultrapure water; no viability was reported
*Microcystis* (7 × 10^5^ cells/mL)	Lab	3, 4, 5	1, 2, 5, 10, 20, 30, 60	min. 2.8 max. 104	Limited impact	<5% (CT 4)	25% degradation (CT 2.8) Complete degradation (CT 104)	[37,68]	Ultrapure water
*Microcystis* (2 × 10^6^ cells/mL)	Lab	0.5 0.7 1.5	5, 11, 50, 60, 120	min. 2.5 max 180	-	<5% (CT 180)	10% degradation 40% increase in released	[69]	Lake water; no CT reported
*Microcystis* (10^6^ cells/mL)	Lab	0.2, 0.4, 0.8	Range 0–480	min. 12 max. 396	-	18% (at CT 12) 0.1% (at CT 396)	-	[70]	Lake water; no CT reported; no cell count; no toxin
*Microcystis* (10^6^ cells/mL)	Lab	1, 2, 4, 8	1, 2, 4, 8, 16, 32, 60	HV min. 0.98 max. 361 LV min. 0.98 max 200	-	HV 95–0% (CT > 15) LV 44–0% (CT > 15)	HV CT↑—degradation↑ Complete (CT 108) CT↑—degradation↑ > 50% release CT > 7 > 62% degradation at highest CT	[71]	Ultrapure water; two viability range
*Microcystis* (1 × 10^6^ cells/mL) (2 × 10^6^ cells/mL)	Lab	1, 2, 4, 8	1, 2, 4, 8, 16, 32, 60	DV min. 3.8 max 356 MA min 3.7 max 293	>95% reduction (CT > 13.3) >95% reduction (CT > 11.9)	No cell viability after oxidation	Same as cell death	[72]	Ultrapure water; two stage of life
*Microcystis*-Colony (10^5^ cells/mL)	Lab	0.3, 0.5, 1, 2	Range 0–20 min	min. 0.97 max. 52	-	Depends on colony size (0–95%)	Release and degradation Colony-size-dependent	[73]	Lake water; different colony size; no cell count
Natural bloom	Field	Cl_2_/DOC: 0.05–3.6	0–20 min	min. 0.15 max 6.8	>80% increase (CT 6.8)	88% reduction	Complete release CT:4 (Cl_2_/DOC: 0.3)	[74]	No CT provided; CT estimated; Chl-a measured as cell damage surrogate
Natural bloom US: (3 × 10^6^ cells/mL) - *Planktothrix* *agardhii* Canada: (3 × 10^5^ cells/mL) - *D. spiroides*	Field	Cl_2_/DOC: 0.05, 025, 0.15, 0.1, 1	0–20 min	US min 0.13 max 15 CA min 0.3 max 21	-	Complete degradation	Complete degradation CT 11 (US), CT 7.5 (CA)	[75]	No cell viability; no cell count; Chl-a measured as cell damage surrogate
Natural bloom (3.3 × 10^5^ cells/mL) *D. spiroides* (5.4 × 10^4^ cells/mL) *M. aeruginosa*	Field	0.2, 0.6	0–120 min	min 0.15 max 3.84	min. CT 5% decrease max. CT 34% decrease	min CT: 82% max CT:55%	CT 3.84: 23% decrease	[76]	Soft chlorination (low dose)
Natural bloom	Field	2, 5	0–60 min	min 1.14 max 14.8	min. < 5% reduction max. > 50% reduction	-	2 mg, CT 10, >200% release 5 mg, CT 20, >200% release	[77]	No cell viability

**Table 2:** Summary of the literature on the impact of pre-ozonation on the cyanobacteria (cultured and natural bloom).

Dominant Cyanobacteria (Cell Density)	Lab/Field	O_3_ Dose (mg/L)	Contact Time (min)	CT (mg min/L)	Cell Count Reduction %	Cell Viability % (for CT)	Toxins	Reference	Comment
*Microcystis* (2 × 10^6^ cells/mL)	Lab	1 2	-	min. 12 max 16	-	CT > 54, complete loss	CT = 12 complete degradation	[66]	Saline solution; exact dose and contact time were not provided; no residual; CT evaluation weak
*Microcystis* (7 × 10^5^ cells/mL)	Lab	2, 4, 6	5	min. < 0.22 max. 2.29	-	Min CT: 50% Max CT: 8.5%	>100% release (high CT) 50% degradation	[37,68]	Ultrapure water
*Microcystis* *D. flos-Aquae* (2.5 × 10^4^ cells/mL (1.5 × 10^5^ cells/mL)	Lab	0.5, 2, 4	0.5–10	min. < 0.2 max. 22	32% for 2 mg/L 41% for 4 mg/L	Complete loss, CT < 0.2	-	[64]	Ultrapure; no flow cytometry
*Microcystis* (2 × 10^5^ cells/mL) *Oscillatoria* (2800 cells/mL) *Lyngbya* sp. (1600 cells/mL)	Lab	0.63–5	24 h	min. 0.5 max 17	100% reduction (CT 0.5)	Complete loss, (CT > 2)	-	[78]	River water; Chl-a measured as cell damage surrogate; no toxin measurement
*Microcystis, Dolichospermum* (4 × 10^5^ cells/mL)	Lab	0.5, 1, 2	5, 10	max. 2.5	>95% reduction	Complete loss	-	[79]	Natural water; no toxin measurement
*Microcystis*, *Dolichospermum* (1.2 × 10^5^–2 × 10^6^ cells/mL)	Field	2, 3, 4, 5	0–10	min. 1.4 max 16.8	75% reduction (CT 16.8)	CT 3.2: 45% CT 16.8: 15%	CT < 2, more than 100% release	[65]	Natural bloom
Natural bloom US (3 × 10^6^ cells/mL)—*Planktothrix agardhii* CA (3 × 10^5^ cells/mL)— *D. spiroides*	Field	O_3_/DOC: 0.05—0.75	0–20	US-min. 1.5 max. 3 CA-min 0.2 max. 4.1	-	-	>80% degradation CT 4.1(CA)	[75]	No cell viability; no cell count; Chl-a measured as cell damage surrogate
Natural bloom (3.3 × 10^5^ cells/mL) *D. spiroides* (5.4 × 10^4^ cells/mL) *M. aeruginosa*	Field	0.1, 0.3	0–10	max: 0.86	max CT 14% decrease	max CT: 79%	14% degradation No release	[76]	Soft ozonation (low dose)

**Table 3:** Summary of the literature on the impact of potassium permanganate on cyanobacteria (cultured and natural bloom).

Dominant Cyanobacteria (Cell Density)	Lab/Field	KMnO_4_ Dose (mg/L)	Contact Time (h)	CT (mg min/L)	Cell Count Reduction %	Cell Viability % (for CT)	Toxins	Reference	Comment
*Microcystis* (2 × 10^6^ cells/mL)	Lab	1–2	-	min. 15 max. 600	-	min. CT: 60%, CT > 60: complete loss	CT: 30 Complete dissolved degradation	[66]	Saline solution; exact dose and contact time were not provided; no residual; CT evaluation weak
*Microcystis* (7 × 10^5^ cells/mL)	Lab	1, 5, 10	0.25–7	min. 28.7 max. 2642	14% cell number reduction (CT max)	CT 2600: complete loss	Release at CT > 70 Complete degradation CT 2600	[37,68]	Ultrapure water
*Microcystis, Dolichospermum* (4 × 10^5^ cells/mL)	Lab	2, 5	20	max. 456	10% reduction at highest CT	CT 456: 18% viability	-	[80]	Natural water; no toxin measurement
*Microcystis* Bloom from Lake Erie	Lab Field	0.5–8	1–5	min. 120 max. 1920	-	Cell, CT 1920: 2% Bloom, CT 1920: 40%	-	[81]	No cell count and toxin; no CT; CT with lower doses was unable to decrease viability

**Table 4:** Summary of the literature on the impact of hydrogen peroxide on cyanobacteria (cultured and natural bloom). h: hour, d: day.

Dominant Cyanobacteria (Cell Density)	Lab/Field	H_2_O_2_ Dose (mg/L)	Contact Time	CT (mg h/L)	Cell Count Reduction %	Cell Viability % (for CT)	Toxins	Reference	Comment
*Microcystis* (3.7 × 10^6^ cells/mL)	Lab	3.4, 17	4 h, 2 d, 4 d	min. 13.6 max. 1632	min. CT: 8% reduction max. CT: 89% reduction	K^+^ release min. CT: 81% max. CT: 5%	CT > 816 26% MC release	[82]	K release as a surrogate for cell damage; no CT provided
*Microcystis* (7 × 10^5^ cells/mL)	Lab	10.2, 51, 102	0.1 d–7 h	min. 189.3 max. 17,678	Limited change	min. CT: 86% CT 4770: 7%	No release, CT 364: >95% degradation	[37,68]	Ultrapure water
*Pseudanabaena* (10^7^ cells/mL)	Lab	3, 5, 10, 20	2 h, 4 h, 8 h, 2 d, 4 d	min. 6 max. 960	min. CT: No change max. CT: >90% reduction	CT 120: 2%	-	[83]	Reservoir water; no toxins
*Microcystis* (6 × 10^6^ cells/mL)	Lab	1–15	0.1 d–7 d	min. 2.4 max. 2520	CT 1680: 95% reduction	max. CT 3% viability	CT > 1512, 82% degradation	[84]	Culture; no CT provided
*Microcystis, Dolichospermum* (4 × 10^5^ cells/mL)	Lab	5, 10	6 h	min. 13.9 max. 96.1	<5% reduction	min. CT: 39% max. CT: 30%	-	[79]	Natural water
Natural bloom: (3.3 × 10^5^ Cells/mL) *D. spiroides:* (5.43 × 10^4^ cells/mL) *M. aeruginosa*	Field	10	6 h–1 d	min. 47 max. 140.7	max. CT 52% reduction	min. CT: 60% max. CT: 40%	No release max.; 15% MC degradation	[76]	-

### 3.2. Chlorination

**Figure 3:**
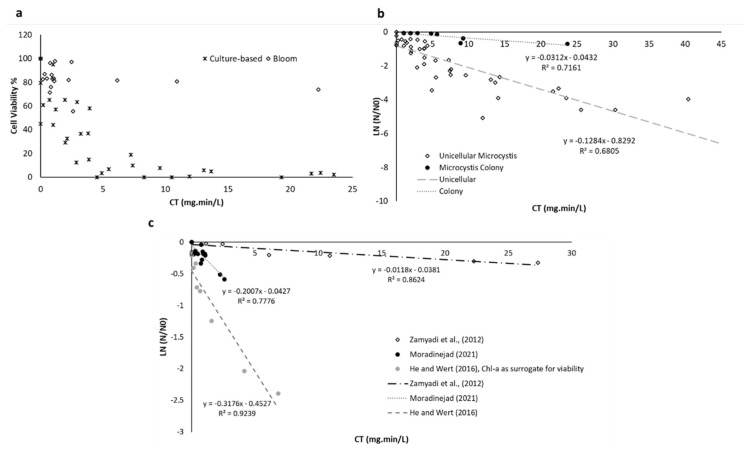
(**a**) Comparison of the cell viability results of cultured-based *Microcystis* and *Dolichospermum* [37,68,71,72,85] and natural cyanobacterial blooms [74,76,77] following pre-chlorination. [74] used Chl-a as a proxy for cell viability. (**b**) Cell viability experimental data and fitted model of unicellular [37,68,71,72,85] and colonial *Microcystis* [73] following pre-chlorination. (**c**) Cell viability experimental data and fitted model for three different cyanobacterial blooms following pre-chlorination.

Figure 3 is a reconstructed graph from the cell viability results following pre-chlorination based on the oxidant exposure (CT). Parameters such as background water quality (e.g., pH and dissolved organic carbon (DOC)) which have an impact on the oxidant demand are included in the CT concept. Therefore, a comparable level of damage should be found by comparing cell viability results using oxidant exposure (CT) for lab-cultured and natural blooms. Figure 3 demonstrates that at the same level of chlorine exposure, the natural bloom is more resistant to pre-chlorination as compared to lab-cultured cells. In other words, lab-cultured studies are not representative of natural bloom pre-chlorination. Fan, et al. [73] reported that the level of cell damage and toxin release depends on the colony size. Figure 3b shows the cultured-based studies fitted with the Chick–Watson equation. Although the results from different unicellular studies are aligned with each other, the colonial *Microcystis* chlorination shows a different cell damage rate. This could be related to the agglomeration of cyanobacteria cells and increasing the mucilage sheath in colonial cyanobacteria [73]. Despite using the CT calculation to compare the results, Figure 3c demonstrates different cell damage rates for each study of real bloom after chlorination, and the same level of chlorine exposure may not result in the same level of cell damage. Figure 3c shows that cyanobacterial bloom oxidation could be site- and bloom-specific, depending on the agglomeration, cyanobacteria (bloom) stage of life, and metabolic functions. Higher cell damage following pre-oxidation (especially with higher CTs) can lead to higher cyanotoxin release, which cannot be removed during conventional treatment. Soft chlorination showed cell damage by up to 45% and total microcystin (MC) degraded by up to 23%, while no cyanotoxin release was observed [76]. In addition, soft chlorination may cause lower disinfection by-products as a lower oxidant concentration is used in this approach.

### 3.3. Ozonation

**Figure 4:**
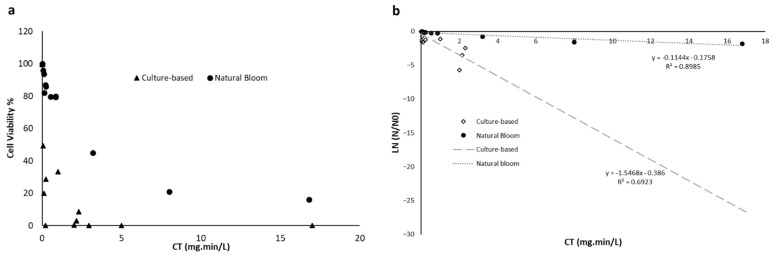
(**a**) Comparison of the cell viability results of cultured-based *Microcystis*, *Dolichospermum*, *Oscillatoria*, *Lyngby asp.* [37,64,68,78,79], and natural blooms [65,76] following pre-ozonation. (**b**) Cell viability experimental data and fitted model for cultured-based and natural bloom samples following pre-ozonation.

Figure 4 shows the impact of pre-ozonation on cyanobacteria cell damage for cultured-based and natural bloom studies. Figure 4a demonstrates lower cyanobacteria cell damage for a specific ozone exposure for natural blooms as compared to the lab-cultured cyanobacteria. The model fit results (Figure 4b) show a higher cell damage rate for the lab-cultured cyanobacteria in comparison to the natural bloom. As per soft oxidation, soft pre-ozonation was reported to cause up to 21% of cell damage and 14% of MC degradation, while no MC release was observed simultaneously [76]. Such an observation implies the effectiveness of soft pre-ozonation to damage the cells without cyanotoxin release.

### 3.4. Potassium Permengeanate

**Figure 5:**
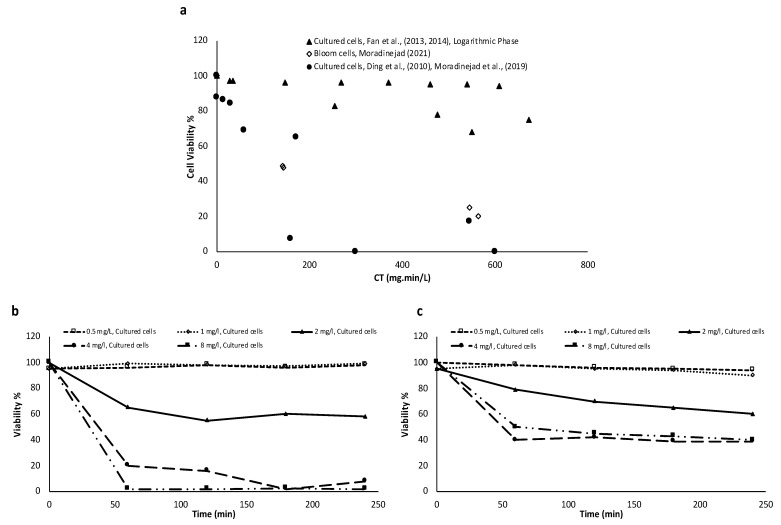
(**a**) Comparison of the cell viability results of cultured-based and natural bloom samples for different studies following potassium permanganate peroxidation: *Microcystis* [37,68], *Microcystis*, *Dolichospermum* [66,79], and natural blooms [76]. (**b**) Comparison of the cell viability results of cultured-based samples (*Microcystis*) following potassium permanganate peroxidation adapted from [81]. (**c**) Comparison of the cell viability results of natural bloom samples (from Lake Erie) adapted from [81].

Figure 5 demonstrates that the viability loss of the lab-cultured studies harvested in the logarithmic phase is lower than those that harvested in the stationary phase. This observation implies the impact of the cyanobacteria stage of life on pre-oxidation efficiency. A comparison of the cell viability results of the lab-cultured with natural bloom studies following potassium permanganate pre-oxidation confirms the higher resistance of real cyanobacterial bloom cells (Figure 5). In addition, the degradation rate constant of dissolved MCs was higher than that released by MCs for high potassium permanganate doses [36,81].

### 3.5. Hydrogen Peroxide

**Figure 6:**
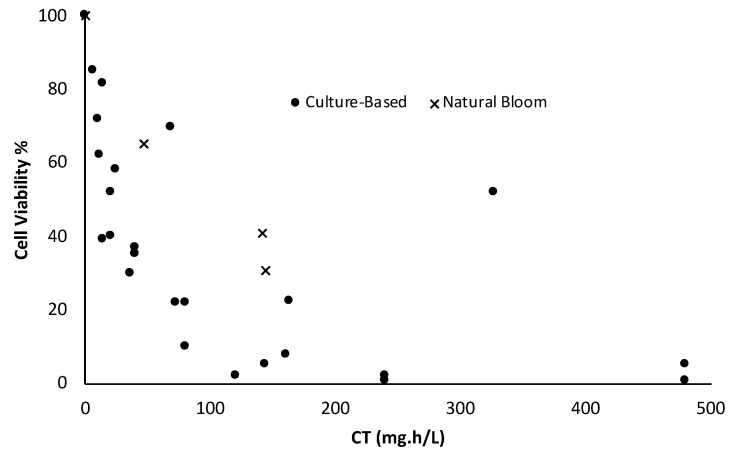
Comparison of the cell viability results of cultured-based cyanobacterial cells (Microcystis, Pseudanabaena) [37,68,83,84] and natural bloom cells [76,86] after oxidation by hydrogen peroxide.

Matthijs, et al. [86] reported that a concentration of 2 mg/L H_2_O_2_ was able to decrease cyanobacteria (natural bloom) by two logs within 3 days. In addition, cyanobacteria remained at a low abundance level for 7 weeks following H_2_O_2_ addition. Figure 6 demonstrates that natural blooms are more resistant to H_2_O_2_ than the lab-cultured cyanobacteria, as observed for other oxidants (Figure 2, Figure 3, Figure 4 and Figure 5). Foo, et al. [87] reported that the impact of H_2_O_2_ on cyanobacteria is dependent on the residual concentration (C) and contact time (T). In addition, the authors concluded that toxic and non-toxic *Microcystis aeruginosa* are impacted by H_2_O_2_ with the same trend. Zhou, et al. [84] stated that a low dose of H_2_O_2_ (<5 mg/L) would have a low and recoverable impact on the lab-cultured *Microcystis*. On the other hand, the higher the H_2_O_2_ dose (>8 mg/L), the higher necrosis, cell death, and consequent cyanotoxin release. A medium dosage of H_2_O_2_ with low to medium contact time can activate apoptosis-like programmed cell death (AL-PCD) [84]. The cellular energy required for AL-PCD is provided from the transcriptional, biochemical, and structural changes. Zhou, et al. [84] documented the maximum cell death with low MC production by AL-PCD activation. Zamyadi, et al. [17] studied the impact of H_2_O_2_ on blooms and lab-cultured cyanobacteria (*Microcystis aeruginosa*). The results highlighted a delayed impact of H_2_O_2_ on cyanobacteria cells after complete depletion of H_2_O_2_ during stagnation (up to one week) [17]. Chl-a and phycocyanin (PC) fluorescence were significantly declined by 93% and 74% in natural bloom and lab-cultured samples, respectively. Additionally, the lab-cultured results revealed delayed MC release during stagnation [17].

Besides the current oxidants, peracetic acid (PAA) has been used in wastewater treatment facilities as a disinfection alternative for chlorine [88]. Almuhtaram and Hofmann [89] studied the impact of PAA and PAA/UV on cyanobacteria and cyanotoxin removal. The results show that 10 mg/L of PAA with 60 min contact time was able to degrade MC-LR by 80% (3.46 M^−1^ s^−1^ lower reaction rate as compared to HOCl 1.2 × 10^2^ M^−1^s^−1^). In addition, the results elaborated that PAA alone can barely remove cyanobacteria, except at a high dose (10 mg/L) and with lower cyanobacterial cell counts (10^5^ cells/mL).

### 3.6. Considerations on the Impact of Pre-Oxidation on Downflow Processes

The impact of pre-oxidation on downflow processes should also be considered as it may influence the removal of cyanobacteria by coagulation, flocculation, and sedimentation. Previous studies have been reported that pre-oxidation has a positive impact on enhancing cyanobacterial removal through coagulation/flocculation and sedimentation [27,28,31,32,34,90]. Pre-oxidation can cause morphological deformation [82] and changes in the surface charge of the cells, leading to increased cell removal efficiency during coagulation/flocculation [37].

KMnO_4_ increases the binding potential to the coagulant by oxidizing organic matter (extracellular and released cell-bound) to lower molecular weight fractions, as well as forming colloids (by MnO_2_) to be adsorbed to the cyanobacterial cells and forming larger flocs [32,34,81]. Xie, et al. [27] reported that KMnO_4_ exposure (CT: 10 mg min/L, estimated) could increase cyanobacteria cell removal by 22% during coagulation/flocculation. In addition, pre-ozonation with CT: 4, 10, and 20 mg min/L (estimated) led to an increase in cyanobacteria cell removal during coagulation by 14%, 20%, and 24%, respectively [27]. Cyanobacteria cell removal during coagulation was improved in a full-scale DWTP equipped by pre-ozonation systems (CT: 2.52–3.78 mg min/L (estimated)) [43]. Pre-oxidation may cause metabolite release (organic matter and cell-bound cyanotoxins) following cyanobacterial cell damage. Besides the challenge to remove dissolved cyanotoxins, coagulation efficiency can be compromised by high algal organic matter release following pre-oxidation. Xie, et al. [27] showed that due to pre-ozonation with CT > 4 mg min/L (estimated), cyanobacteria cell viability was completely degraded, and consequently, organic matter concertation increased. Further, Barešová, et al. [91] demonstrated that pre-ozonation (CT < 40 mg min/L (estimated)) could interrupt the coagulation (Al/Fe-based) efficiency of DOC removal (in comparison with higher CTs) due to the degradation of high molecular weight algal organic matter to low molecular weight compounds.

It is noteworthy to recall that H_2_O_2_ can have a delayed impact on cyanobacteria and, potentially, cyanotoxin release after complete degradation of the oxidant [17]. This delayed cyanotoxin release should be considered in the downstream processes, as well as in sludge handling.

**Figure 7:**
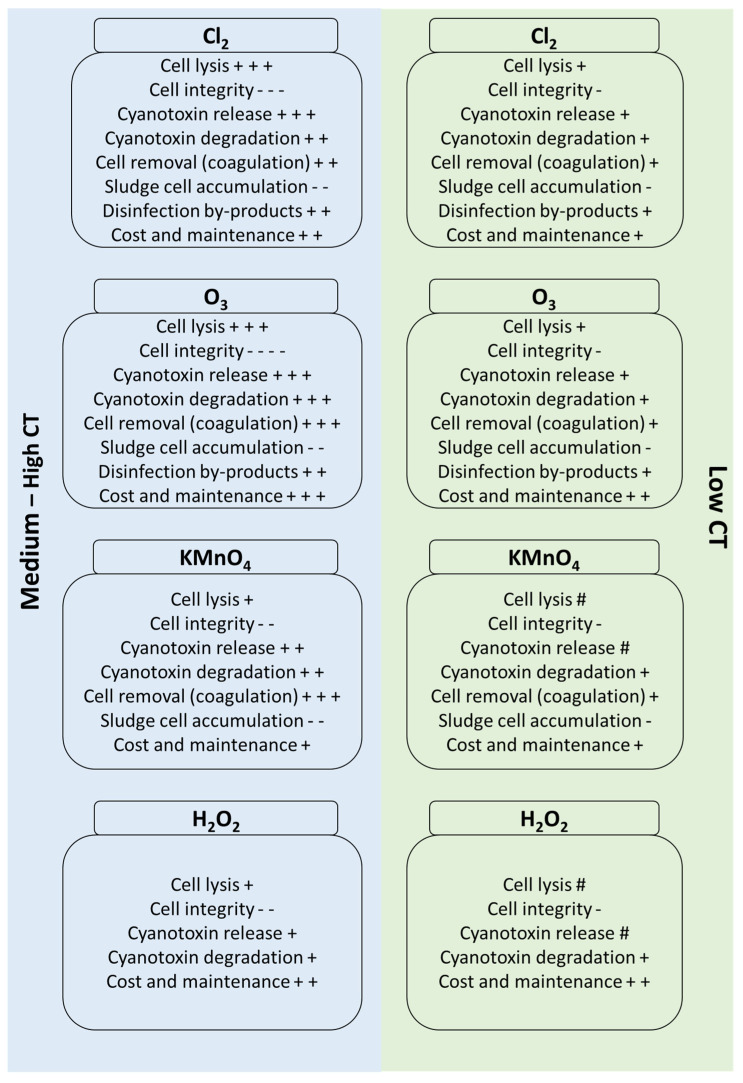
Summary of pre-oxidation (low and medium-high CT) impact on cyanobacteria/cyanotoxins and downflow processes (#: very low impact, +: increase, and -: decrease). Low CT for Cl_2_ = CT < 4 mg min/L, low CT for O_3_ = CT < 1 mg min/L, low CT for KMnO_4_ = CT < 50 mg min/L, and low CT for H_2_O_2_ = CT < 50 mg h/L.

The oxidant exposure must be adjusted to maximize cell damage and cyanobacteria cell removal (directly or after coagulation) and minimize cyanotoxin release and cell accumulation in the sludge, simultaneously. Figure 7 summarizes the pre-oxidation (soft and normal) advantages/disadvantages of cyanobacteria and cyanotoxins during water treatment. In fact, soft pre-oxidation (low CT of Cl_2_ and O_3_) can (1) partially degrade cyanobacteria cells, (2) cause low cyanotoxin release, (3) improve coagulation efficiency to remove cells, and (4) cause low cell accumulation in the downflow processes.

## 4. Sludge Storage, Oxidation, and Handling

Cyanobacteria and cyanotoxins (cell-bound) accumulate in the sludge of clarifiers throughout the flocculation/coagulation/sedimentation processes. This cyanobacteria-laden sludge remains in the sludge holding tank before disposal. In addition, potential options to treat cyanobacteria-laden sludge need to be considered. Furthermore, safe (healthy, both operationally and environmentally) cyanobacteria-laden sludge handling approaches are required.

### 4.1. Fate of Cyanobacteria and Cyanotoxins during Sludge Storage

Several studies (Table 5) demonstrated that cyanobacteria cells could stay viable within 2–12 days in the stored sludge. The loss of viability and consequent cyanotoxin release caused an increase in dissolved cyanotoxin concentrations during sludge storage [13,40,45,46,47,48,49,50,51,52]. However, dissolved cyanotoxins in stored sludge can be adsorbed onto the remained PAC injected into the intake water [56], flocs [50] or it can be biodegraded by cyanotoxin degrader species [58,92].

Besides cell survival potential during sludge storage, some studies have hypothesized that cyanobacteria can also grow in stored sludge [53,54,55]. Water Research Foundation (WRF) and Water Research Australia [53] documented that concentrations of DOC, MC-LR, and cylindrospermopsin in stored coagulated sludge contained *M. aeruginosa* and *C. raciborskii* exceeded the expected concentrations by 4–10-fold based on the cell quota (if all cell-bound metabolites are released) within 7–16 days, respectively. Dreyfus, et al. [55] studied the fate of stored sludge that contained cultured *M. aeruginosa*, *D. circinale*, and *C. raciborskii* within 18 days. The authors demonstrated that DOC, MC-LR, MC-LA, and CYN concentrations increased by up to 5-, 2.2-, 1.2-, and 2.5-fold during storage, respectively. Another investigation on stored sludge containing cultured *M. aeruginosa* and *D. circinale* reported that taxonomic cell counts increased by up to 4.2-fold in sludge stored in a lagoon within 7 days [54]. The authors also reported that the concentrations of cyanobacterial metabolites increased by up to 5 times in the sludge supernatant within 20 days. In the worst case, cyanobacteria could survive by up to 35 days in the stored sludge [54]. Despite the important findings of the previous studies on cell survival and metabolite release during sludge storage, cyanobacterial cell growth during sludge storage is yet to be explored in detail. In these studies, cell and metabolite increase during sludge storage might be due to the cell growth or to either the (1) underestimation of cell quota, (2) increase of metabolite production per cell during storage, or (3) additional cell settling from the supernatant to the sludge during the storage [53,54,55].

Our recent study on the cyanobacteria-laden sludge of a DWTP documented cell depletion, survival, and growth in different sludge samples [58]. Cell growth was observed in four out of eight sludge samples (different sampling dates) stored in the dark for 7–38 days. In the worst-case scenario, taxonomic cell counts increased from 2.7 × 10^6^ to 5.3 × 10^6^ cells/mL within 16 days (96% cell growth). Cell growth was also confirmed by increasing cyanobacterial biomarkers such as the “Pentose phosphate pathway” marker, which is responsible for the heterotrophic growth of cyanobacteria [93].

**Table 5:** Impact of sludge storage on cyanobacteria and cyanotoxins. STX: saxitoxin, PACl: colyaluminium chloride, CTSAC: Chitosan-aluminum chloride.

Initial Characteristics of Cyanobacteria/ Coagulation/Sedimentation Process	Initial Condition of Cyanobacteria and Cyanotoxins in the Stored Sludge	Observation	Reference
Cultured *M. aeruginosa* (1 × 10^6^ cells/mL) (Jar test, 70 mg/L alum)	8 × 10^6^ cells/mL, 2500 µg MC-LR/L	Cell survival (2 days); cell lysis and cyanotoxin release (2 days); degradation of dissolved cyanotoxins (8–10 days)	[13]
Cultured *D. circinale* and *C. raciborskii* (1.0 × 10^5^ cells/mL) (Jar test, 40 mg/L alum)	Sludge supernatant: *D. circinale*: 1300 cells/mL STX: 0.4 µg/L	Cells remained viable up to 7 days; cell lysis and toxin release within 3 days	[45]
Cultured *M. aeruginosa* (2 × 10^6^ cells/mL) (Jar test, 15 mg/L AlCl_3_)	18 µg/L dissolved MCs	Cell lysis and cyanotoxin release after 6 days	[40]
Cultured *M. aeruginosa* (1 × 10^6^ cells/mL) (Jar test, 4 mg/L PACl-optimum dose)	20 µg/L dissolved MCs	Cell lysis and cyanotoxin release within 6–12 days	[46]
*Microcystisflos aquae* (5.2 × 10^5^ cells/mL) (Jar test, 100 mg/L alum)	Sludge supernatant: MC-RR, MC-YR: < 2 µg/L	Cell survival (5 days); cell lysis and cyanotoxin release (5–10 days); degradation of dissolved cyanotoxins (up to 15 days)	[62]
Cultured *M. aeruginosa* (1 × 10^6^ cells/mL) (Jar test, 15 mg/L ALCl_3_, 4 mg/L PACl)	−0.9 bar vacuum pressure for dewatering the sludge 23 µg/L total MCs	Cell lysis and cyanotoxin release within 4–6 days; optimum sludge storage time for AlCl_3_ and PACl was suggested to be 4 and 2 days, respectively.	[47]
Cultured *M. aeruginosa* (1 × 10^6^ cells/mL) (Jar test, 0–70 mg/L FeCl_3_)	~1 µg/L dissolved MCs	Cell lysis and cyanotoxin release (2–8 days); degradation of dissolved cyanotoxins (> 10 days)	[48]
Myponga reservoir Cultured *M. aeruginosa* (2.3 × 10^5^ cells/mL) Cell-bound MC-LR: 4.7 µg/L Dissolved MC-LR: 2.0 µg/L (Jar test-80 mg/L alum)	Sludge supernatant after 1 day storage: Cells: 4300 cells/mL Cell-bound MC-LR: 0.5 µg/L Dissolved MC-LR: 2.5 µg/L	Cell survival (4 days); cell lysis and cyanotoxin release (4–7 days); degradation of dissolved cyanotoxins (> 4 days)	[53]
Myponga reservoir Cultured *M. aeruginosa* (3.1 × 10^5^ cells/mL) DOC: 10.1 mg/L Cell-Bound MC-LR: 5.0 µg/L Dissolved MC-LR: 2.9 µg/L (Jar test-80 mg/L alum)	Sludge supernatant after 1 day storage: DOC: 5.2 mg/L Cell: 2760 cells/mL Cell-bound MC-LR: <DL Dissolved MC-LR: 4.0 µg/L	Cell growth (within 7–16 days) confirmed by DOC and MC-LR cell quota	
Myponga reservoir Cultured *C. raciborskii* (3.1 × 10^5^ cells/mL) DOC: 10 mg/L Cell-bound CYN: 2.5 µg/L Dissolved CYN: 0.7 µg/L (Jar test-80 mg/L alum)	Sludge supernatant after 1 day storage: DOC: 6.0 mg/L Cell: 7080 cells/mL Cell-bound CYN: 1.0 µg/L Dissolved CYN: 0.8 µg/L	Cell growth (within 7–23 days) confirmed by DOC and CYN cell quota	
River Murary Cultured *C. raciborskii* (3.1 × 10^5^ cells/mL) DOC: 8.63 mg/L Cell-bound CYN: 2.7 µg/L Dissolved CYN: 0.3 µg/L (Jar test-80 mg/L alum)	Sludge supernatant after 1 day storage: DOC: 4.9 mg/L Cell: 4140 cells/mL Cell-bound CYN: 0.3 µg/L Dissolved CYN: 0.9 µg/L	Cell growth (within 15–23 days) confirmed by DOC and CYN cell quota	
Cultured *M. aeruginosa* (1 × 10^6^ cells/mL) (Jar test- 15 mg/L AlCl_3_, 50 mg/L FeCl_3_, 15 mg/L PAFC)	20 µg/L dissolved MCs 1–4.2 mg/L dissolved polysaccharides 4 mg/L chla	Cell lysis and toxin release (2–10 days)	[49]
Cultured *M. aeruginosa* (2 × 10^6^ cells/mL) (Jar test, 2.6 mg/L chitosan- 7.5 mg/L AlCl_3_ (CTSAC)	9 µg/L dissolved MCs (after coagulation) 18 µg/L dissolved MCs (without coagulation); the difference is due to adsorption in CTSAC	Toxin release (0–4 days); degradation of dissolved cyanotoxins (6–10 days)	[50]
*M. aeruginosa, D. circinale, C. raciborskii* (3.0 × 10^5^ cells/mL) (Jar test, 80 mg/L Alum)	Sludge supernatant after 1 day storage: DOC: 5.2–6.5 mg/L Cell: 2162–7080 cells/mL Cell-bound MC-LR: <0.5 µg/L Dissolved MC-LR: 2.5–4.0 µg/L Cell-bound CYN: 1.0 µg/L Dissolved CYN: 0.8 µg/L	Increased DOC, MC-LR, MC-LA, and CYN to higher the expected values (hypothesis: increase of the metabolite production, cell growth or both)	[55]
*M. aeruginosa* and *D. circinale* (8.6 × 10^4^ cells/mL) (Jar test, 80 mg/L Alum)	Non-coagulated sludge: 5.0 × 10^6^ cells/mL Coagulated sludge: 5.4 × 10^5^ cells/mL	Cell survival (up to 35 days); 4.2× increase in cell counts in the sludge lagoon within 7 days; increased metabolites to higher the expected values (up to 5×); increased cell counts in the sludge (hypothesis: cell growth, additional settling, or both)	[54]
Cultured M. aeruginosa × 10^5^ cells/mL) (Jar test, 15 mg/L AlCl_3_, 50 mg/L FeCl_3_, 15 mg/L PAFC)	1 µg/L dissolved MCs	Cell lysis and toxin release (4–6 days); degradation of dissolved cyanotoxins (6–10 days)	[51]
Cultured *Oscillatoria sp.* (1.0 × 104 cells/mL) (Jar test, 5 and 10 mg/L PAFC)	1.0 mg/L chla 2.3 µg/L cell-bound protein 8.6–11.4 µg/L dissolved CYN	Increase in chla level after 4 days, suggesting cell growth; loss of cell integrity after 2 days, while cells remained viable up to 8 days; increase in dissolved CYN, showing toxin release within 4 days	[94]
Cultured *C. raciborskii* (1 × 10^6^ cells/mL at late exponential phase) (Jar test, 10 mg/L PAFC)	1.1 µg/L dissolved CYN 2 mg/L cell-bound protein	Cell lysis and toxin release after 6 days; degradation of dissolved cyanotoxins after 10 days	[52]
n/a	Sludge of a DWTP containing natural cyanobacterial blooms stored for 7–35 days in the darkness (8 samples). 0.7 × 105–5.6 × 106 cells/mL 25–7130 ng/L cell-bound MCs 38–349 ng/L dissolved MCs	Cell growth in 4/8 samples after 9–35 stagnation days; cell death in the rest 4/8 samples; degradation of dissolved cyanotoxins after 8 days	[58]

### 4.2. Cyanobacteria-Laden Sludge Treatment

A summary of studies on the treatment of cyanobacteria-laden sludge is presented in Table 6. The available data demonstrated that sludge oxidation could not completely remove cyanobacteria cells and metabolites from the sludge [57,59]. Sludge is often stored after oxidation, while its supernatant can be recycled to the head of the DWTP. Thus, the impact of oxidation on sludge storage should be investigated. Recent findings showed no remarkable benefits in sludge oxidation followed by sludge storage as compared to only sludge storage [59]. The maximum additional taxonomic cell count decreased by a combination of oxidation (KMnO_4_ or H_2_O_2_) and storage was 32% as compared to storage only. However, oxidation/storage could cause a remarkable cell growth (by up to 145%) and toxic gene copy numbers of *mcyD* increase (by up to 13.0×) in some sludge samples [59]. This phenomenon can be attributed to gene expression regulation due to the presence of oxidative stresses [58,59,95,96]. Similarly, sludge oxidation could not completely remove cyanobacteria and cyanotoxins from the supernatant sludge [59]. Finally, the costs and by-product formation during the oxidation of organic-matter-rich sludge should be considered [58].

### 4.3. Sludge Handling Challenges

In general, the sludge supernatant is recycled to the head of the DWTP or is discharged into the source [13,56,97]. The solid phase either can be transferred to the WWTP or is applied for landfilling [98,99,100]. Less environmentally friendly approaches such as untreated residual discharge into lakes or ponds can be also applied in some circumstances [101]. The re-use of DWTPs’ residuals is growing [99,102,103,104]. However, an investigation demonstrated that the half-life of MC analogs varies from 8 to 18 days in soil [105]. Since there is a risk of soil and groundwater contamination, landfill and field applications of cyanobacteria-laden sludge should be avoided. Overall, cyanobacteria-laden sludge should be treated before disposal either in situ or via sending it to wastewater treatment plants.

Flocs may have a protective role during sludge storage for *M. aeruginosa* [40,46,47,48,52]. In contrast, Li, et al. [52] documented that polyaluminium ferric chloride (PAFC) can stimulate the lysis of *C. raciborskii* and CYN release by up to 94% during sludge storage. This may occur in the sludge and lead to cyanotoxin release. However, all studies have been conducted in laboratory conditions and on cultured-based cyanobacteria. In fact, due to complex parameters such as the presence of various cyanobacterial cells in various forms (aggregated, multicellular), ages, and viabilities, the design of such experiments in full scale is complex.

Stresses such as oxidation and storage can shift cyanobacterial communities towards resistant genera (e.g., *Microcystis* and *Aphanocapsa*), which can produce MCs [56,58,59]. Thus, the survival probability of MC producer species can increase during sludge oxidation or storage. The fate of cyanotoxins in the sludge is complex due to the simultaneous occurrence of various phenomena such as cell survival, growth, lysis, cell-bound cyanotoxin release, and released cyanotoxin degradation [53,54,55,56,59,106,107]. Based on the increased risk of cell lysis and cyanotoxin release during sludge storage, some studies have suggested that cyanobacteria-laden sludge should be disposed of prior to 4 days to avoid metabolite release [47,51,108]. However, these studies only focused on metabolite release and not on cell survival/growth phenomena. Additionally, the possibility of sludge disposal can be a technical and financial challenge in large DWTPs.

## 5. Decision Framework to Manage Cyanobacteria and Cyanotoxins in Drinking Water Treatment Plants

### 5.1. Framework Basis

Since cyanobacterial cells and their associated metabolites, including cyanotoxins, as well as taste and odor agents such as geosmin and 2-Methylisoborneol (MIB), affect water and sludge quality, monitoring should be applied for the evaluation of the water treatment chain and sludge handling.

Microscopy taxonomic cell count techniques have been widely applied to evaluate the water and sludge in previous studies [12,18,43,44,56,77]. Previous cyanobacterial monitoring guidelines were prepared based on taxonomic cell counts and biovolumes [109,110,111]. A recent study suggested 0.3 mm^3^/L biovolumes as the vigilance level [109]. However, bias related to human error [112], the negative impact of Lugol’s iodine on biovolumes [113], cell underestimation/overestimation due to the presence of aggregated cells [114], and the presence of debris and sediments, especially in the sludge samples [56], may affect the results. More importantly, the significant time required for taxonomic cell counts is a major barrier in using them for a real-time/practical approach.

In situ fluorometry using on-line probes is a compromising technique for measurement of PC based on relative fluorescence units (RFU) in water resources [18,115,116,117,118,119]. However, the correlation between RFU and biovolume is complex and site-specific [119,120,121]. Previous investigations have reported that various RFUs ranging from 0.7 to 1.8 could correlate with a 0.3 mm^3^/L biovolume in different sources and bloom events [116,119]. Therefore, it is recommended to perform a correlation between on-line probe readings (RFU) and biovolumes for each water resource. It is noteworthy that the limits of detection and quantification of on-line probes should be considered [116,118].

MC concentration has been introduced in several guidelines such as those of the WHO (1.0 μg/L MC-LR) [122,123] and Health Canada (1.5 μg/L) [124]. Geosmin and MIB negatively affect water quality, raise complaints about taste and odor, and decrease the public’s confidence about the treated water safety [125,126,127,128,129]. Thus, they should be monitored throughout the treatment chain and the recycled sludge supernatant. Using enzyme-linked immunoassay (ELISA) tests for cyanotoxins measurement has been accepted for cyanotoxin monitoring [44,130,131,132]. The reported thresholds for geosmin and MIB are 1.3–4.0 ng/L and 6.3–15 ng/L, respectively [133,134,135]. Since the taste and odor agents are not harmful, but increase complaints and concerns about the water quality [126,127,129,134], olfactory detection can be considered for monitoring and detection [128,136].

### 5.2. Decision Framework

**Figure 8:**
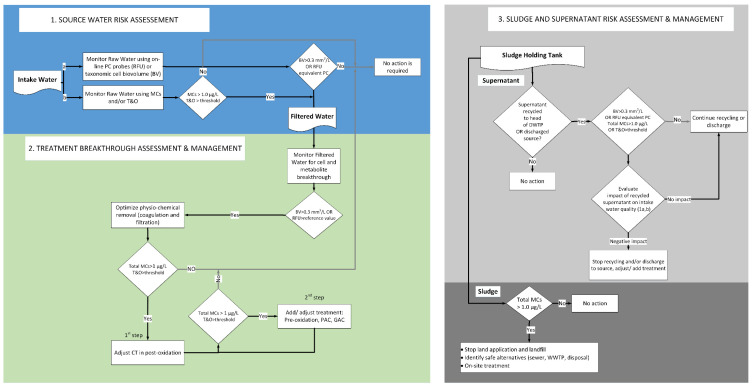
Decision framework for cyanobacterial bloom management in drinking water treatment utilities.

A decision framework to manage cyanobacteria and cyanotoxins in DWTPs is presented in Figure 8. The objective of this framework is to minimize cell breakthrough and accumulation throughout DWTPs and sludge. The three steps, (i) source water risk assessment, (ii) treatment breakthrough assessment and management, and (iii) sludge and supernatant risk assessment and management, should be taken for cyanobacteria and cyanotoxin control in DWTPs and sludge. This framework can help water utilities to understand appropriate approaches/strategies against cyanobacteria and cyanotoxins in DWTPs.

Overall, taxonomic cell counts, MCs, and taste and odor agents should be monitored in the (i) intake water, (ii) treatment chain, and (iii) sludge supernatant. These points are subjected to cyanobacteria and cyanotoxin accumulation, leading to a negative impact on water quality.

The optimization of conventional processes may include coagulant dose adjustment, applying aid-flocculants, and lowering the sedimentation and filtration rate during cell breakthrough [12,137,138,139]. Secondary barriers such as pre-oxidation, PAC injection, and GAC, in case of metabolite breakthrough, should be applied [15,25,39,65,140,141,142,143,144]. The impact of supernatant recycling or discharging on the source/intake water quality should be considered during toxic cyanobacterial blooms. Supernatant treatment may be required in the presence of cyanotoxins or taste and odor agents. MC concentration levels should be monitored in the sludge (solids) in case of landfilling or land application. In the case of elevated concentrations of MCs, sludge treatment is required.

## 6. Conclusions

○ Using the exposure unit (CT) is recommended for cyanobacteria and cyanotoxins oxidation studies, rather than using dose or contact time individually.
○ Regardless of the oxidant type, lab-cultured studies cannot depict the complete picture of natural cyanobacterial bloom behavior during oxidation and may overestimate the oxidation efficiency. In addition, cyanobacterial bloom oxidation is site- and bloom-specific, which could be related to the level of agglomeration, cyanobacteria (bloom) stage of life, and metabolic functions.
○ Soft pre-chlorination and pre-ozonation can compromise cell viability with no or limited cyanotoxin release. Overall, soft pre-oxidation may cause lower disinfection by-products compared to normal pre-oxidation.
○ The cyanobacteria in stored sludge can not only survive, but also grow and release cyanotoxins, even in the dark. Although dissolved cyanotoxins can be degraded during sludge storage, the potential risk of growth and cyanotoxin release should be considered. In fact, the cell growth/depletion in stored sludge is complex and not easy to predict. Therefore, the worst-case scenario should be considered during sludge handling.
○ Due to the low efficacy of sludge oxidation as compared to only stored sludge, as well as the occurrence of cell growth, and gene expression regulation during oxidation/storage, oxidation cannot be a reliable approach in sludge treatment and management.
○ Management of cyanobacteria and cyanotoxins in sludge should be initiated with the minimization of cyanobacteria and cyanotoxin accumulation throughout DWTPs.
○ To control the negative impacts of cyanobacterial accumulation in DWTPs, recycling sludge supernatant to the head of the DWTPs should be regulated during cyanobacterial seasons. Suitable treatment and disposal approaches should be set into guidance and regulations for sludge-containing cyanotoxins.

## Figures and Tables

**Table 6:** Data of cyanobacteria-laden sludge treatment. MIB: 2-Methylisoborneol.

Source of Sludge	Scale	Treatment Agent/Dosage	Contact Time	Initial Conditions	Cell Count Reduction	Metabolite Reduction	Reference
Sludge thickener	Laboratory	3 mg/L KMnO_4_	2 h	5.0 × 10^4^ cells/mL *Pseudanabaena*	>95%	-	[57]
	Laboratory	10–100 mg/L PAC	1 h	100/L MIB	-	42–100% MIB	
	Full-scale	10 mg/L KMnO_4_	15 h (max.)	4.3 × 10^5^ cells/mL (natural blooms)	13–98% total and *Pseudanabaena* cell counts	-	
	Full-scale	10 mg/L KMnO_4_ 20 mg/L PAC	KMnO_4_: 24–72 h PAC: 1 h	3.7 × 10^5^ cells/mL 120 ng/L MIB (natural blooms)	40–52% in total and *Pseudanabaena* cell counts	20–22% MIB	
Sludge holding tank	Laboratory	5 mg/L KMnO_4_	60 min	2.3–2.7 × 10^6^ cells/mL 63–161 ng/L MCs (natural blooms)	46–55% total cell counts	0.3–24% MCs	[59]
		10 mg/L KMnO_4_			59–62% total cell counts	2–32% MCs	
		10 mg/L H_2_O_2_	24 h		58% total cell counts	27% MCs	
		20 mg/L H_2_O_2_			77% total cell counts	41% MCs	
	Full-scale (shock oxidation)	10 mg/L KMnO_4_	24–72 h	2.4 × 10^6^ cells/mL 88–1083 ng/L MCs (natural blooms)	24–43% total cell counts (31% cell count increase after 48 h in one sample)	MCs: 3–25% decrease in one sample 37–589% increase in one sample	

## References

[1] Churro C., Dias E., Valério E. (2012). Risk assessment of cyanobacteria and cyanotoxins, the particularities and challenges of *Planktothrix* spp. monitoring. Novel Approaches and Their Applications in Risk Assessment.

[2] Kosten S., Huszar V.L.M., Bécares E., Costa L.S., Donk E., Hansson L.-A., Jeppesen E., Kruk C., Lacerot G., Mazzeo N. (2012). Warmer climates boost cyanobacterial dominance in shallow lakes. Glob. Chang. Biol..

[3] Paerl H.W., Paul V.J. (2012). Climate change: Links to global expansion of harmful cyanobacteria. Water Res..

[4] Chorus I., Fastner J., Welker M. (2021). Cyanobacteria and Cyanotoxins in a Changing Environment: Concepts, Controversies, Challenges. Water.

[5] Mokoena M.M., Mukhola M.S. (2019). Current Effects of Cyanobacteria Toxin in Water Sources and Containers in the Hartbeespoort Dam Area, South Africa. Int. J. Environ. Res. Public Health.

[6] Huisman J., Codd G.A., Paerl H.W., Ibelings B.W., Verspagen J.M.H., Visser P.M. (2018). Cyanobacterial blooms. Nat. Rev. Microbiol..

[7] O’Keeffe J. (2019). Cyanobacteria and Drinking Water: Occurrence, Risks, Management and Knowledge Gaps for Public Health.

[8] Mishra S., Stumpf R.P., Schaeffer B.A., Werdell P.J., Loftin K.A., Meredith A. (2019). Measurement of Cyanobacterial Bloom Magnitude using Satellite Remote Sensing. Sci. Rep..

[9] Akyol C., Ozbayram E.G., Accoroni S., Radini S., Eusebi A.L., Gorbi S., Vignaroli C., Bacchiocchi S., Campacci D., Gigli F. (2021). Monitoring of cyanobacterial blooms and assessing polymer-enhanced microfiltration and ultrafiltration for microcystin removal in an Italian drinking water treatment plant. Environ. Pollut..

[10] Zamyadi A., Glover C.M., Yasir A., Stuetz R., Newcombe G., Crosbie N.D., Lin T.-F., Henderson R. (2021). Toxic cyanobacteria in water supply systems: Data analysis to map global challenges and demonstrate the benefits of multi-barrier treatment approaches. H_2_Open J..

[11] Westrick J.A., Szlag D.C., Southwell B.J., Sinclair J. (2010). A review of cyanobacteria and cyanotoxins removal/inactivation in drinking water treatment. Anal. Bioanal. Chem..

[12] Zamyadi A., Dorner S., Ellis D., Bolduc A., Bastien C., Prévost M. (2013). Species-dependence of *cyanobacteria* removal efficiency by different drinking water treatment processes. Water Res..

[13] Drikas M., Chow C.W.K., House J., Burch M.D. (2001). Using coagulation, flocculation, and settling to remove toxic cyanobacteria. J. Am. Water Work. Assoc..

[14] Szlag D.C., Sinclair J.L., Southwell B., Westrick J.A. (2015). Cyanobacteria and Cyanotoxins Occurrence and Removal from Five High-Risk Conventional Treatment Drinking Water Plants. Toxins.

[15] Newcombe G., Nicholson B. (2004). Water treatment options for dissolved cyanotoxins. Water Supply Res. Technol.-Aqua.

[16] Wert E.C., Rosario-Ortiz F.L. (2013). Intracellular organic matter from cyanobacteria as a precursor for carbonaceous and nitrogenous disinfection byproducts. Environ. Sci. Technol..

[17] Zamyadi A., Greenstein K.E., Glover C.M., Adams C., Rosenfeldt E., Wert E.C. (2020). Impact of hydrogen peroxide and copper sulfate on the delayed release of microcystin. Water.

[18] Zamyadi A., MacLeod S., Fan Y., McQuaid N., Dorner S., Sauvé S., Prévost M. (2012). Toxic cyanobacterial breakthrough and accumulation in a drinking water plant: A monitoring and treatment challenge. Water Res..

[19] Henderson R.K., Parsons S.A., Jefferson B. (2010). The impact of differing cell and algogenic organic matter (AOM) characteristics on the coagulation and flotation of algae. Water Res..

[20] Newcombe G. (2009). International Guidance Manual for the Management of Toxic Cyanobacteria.

[21] Gonzalez-Torres A., Putnam J., Jefferson B., Stuetz R.M., Henderson R.K. (2014). Examination of the physical properties of Microcystis aeruginosa flocs produced on coagulation with metal salts. Water Res..

[22] Pivokonsky M., Naceradska J., Brabenec T., Novotna K., Baresova M., Janda V. (2015). The impact of interactions between algal organic matter and humic substances on coagulation. Water Res..

[23] Merel S., Walker D., Chicana R., Snyder S., Baures E., Thomas O. (2013). State of knowledge and concerns on cyanobacterial blooms and cyanotoxins. Environ. Int..

[24] He X., Liu Y.-L., Conklin A., Westrick J., Weavers L.K., Dionysiou D.D., Lenhart J.J., Mouser P.J., Szlag D., Walker H.W. (2016). Toxic cyanobacteria and drinking water: Impacts, detection, and treatment. Harmful Algae.

[25] Abbas T., Kajjumba G.W., Ejjada M., Masrura S.U., Marti E.J., Khan E., Jones-Lepp T.L. (2020). Recent Advancements in The Removal of Cyanotoxins from Water Using Conventional and Modified Adsorbents—A Contemporary review. Water.

[26] Villars K., Huang Y., Lenhart J.J. (2020). Removal of the Cyanotoxin Microcystin-LR from Drinking Water Using Granular Activated Carbon. Environ. Eng. Sci..

[27] Xie P., Ma J., Fang J., Guan Y., Yue S., Li X., Chen L. (2013). Comparison of permanganate preoxidation and preozonation on algae containing water: Cell integrity, characteristics, and chlorinated disinfection byproduct formation. Environ. Sci. Technol..

[28] Lapsongpon T., Leungprasert S., Yoshimura C. (2017). Pre-chlorination contact time and the removal and control of *Microcystis aeroginosa* in coagulation. IOP Conf. Ser. Earth Environ. Sci..

[29] Petrusevski B., van Breeman A.N., Alaerts G.J. (1996). Effect of permanganate pre-treatment and coagulation with dual coagulants on algae removal in direct filtration. Water Supply Res. Technol.-Aqua.

[30] Chu W., Yao D., Deng Y., Sui M., Gao N. (2017). Production of trihalomethanes, haloacetaldehydes and haloacetonitriles during chlorination of microcystin-LR and impacts of pre-oxidation on their formation. J. Hazard. Mater..

[31] Lin J.L., Hua L.C., Hung S.K., Huang C. (2018). Algal removal from cyanobacteria-rich waters by preoxidation-assisted coagulation-flotation: Effect of algogenic organic matter release on algal removal and trihalomethane formation. J. Environ. Sci..

[32] Naceradska J., Pivokonsky M., Pivokonska L., Baresova M., Henderson R.K., Zamyadi A., Janda V. (2017). The impact of pre-oxidation with potassium permanganate on cyanobacterial organic matter removal by coagulation. Water Res..

[33] Wang H., Masters S., Falkinham J.O., Edwards M.A., Pruden A. (2015). Distribution system water quality affects responses of opportunistic pathogen gene markers in household water heaters. Environ. Sci. Technol..

[34] Xie P., Chen Y., Ma J., Zhang X., Zou J., Wang Z. (2016). A mini review of preoxidation to improve coagulation. Chemosphere.

[35] Zamyadi A., Fan Y., Daly R.I., Prévost M. (2013). Chlorination of *Microcystis aeruginosa*: Toxin release and oxidation, cellular chlorine demand and disinfection by-products formation. Water Res..

[36] Fan J., Daly R., Hobson P., Ho L., Brookes J. (2013). Impact of potassium permanganate on cyanobacterial cell integrity and toxin release and degradation. Chemosphere.

[37] Fan J., Ho L., Hobson P., Brookes J. (2013). Evaluating the effectiveness of copper sulphate, chlorine, potassium permanganate, hydrogen peroxide and ozone on cyanobacterial cell integrity. Water Res..

[38] Moradinejad S., Trigui H., Maldonado J.F.G., Shapiro B.J., Terrat Y., Sauvé S., Fortin N., Zamyadi A., Dorner S., Prévost M. (2021). Metagenomic study to evaluate functional capacity of a cyanobacterial bloom during oxidation. Chem. Eng. J. Adv..

[39] Moradinejad S., Trigui H., Guerra Maldonado J.F., Shapiro J., Terrat Y., Zamyadi A., Dorner S., Prévost M. (2020). Diversity assessment of toxic cyanobacterial blooms during oxidation. Toxins.

[40] Sun F., Pei H.-Y., Hu W.-R., Ma C.-X. (2012). The lysis of Microcystis aeruginosa in AlCl3 coagulation and sedimentation processes. Chem. Eng. J..

[41] Teixeira M.R., Rosa M.J. (2007). Comparing dissolved air flotation and conventional sedimentation to remove cyanobacterial cells of Microcystis aeruginosa. Part II. The effect of water background organics. Sep. Purif. Technol..

[42] Chorus I., Bartram J. (1999). Chapter 6. Situation assessment, planning and management. Toxic Cyanobacteria in Water: A Guide to Their Public Health Consequences, Monitoring and Management.

[43] Zamyadi A., Dorner S., Ndong M., Ellis D., Bolduc A., Bastien C., Prévost M. (2013). Low-risk cyanobacterial bloom sources: Cell accumulation within full-scale treatment plants. J. Am. Water Work. Assoc..

[44] Almuhtaram H., Cui Y., Zamyadi A., Hofmann R. (2018). Cyanotoxins and cyanobacteria cell accumulations in drinking water treatment plants with a low risk of bloom formation at the source. Toxins.

[45] Ho L., Dreyfus J., Boyer J.e., Lowe T., Bustamante H., Duker P., Meli T., Newcombe G. (2012). Fate of cyanobacteria and their metabolites during water treatment sludge management processes. Sci. Total Environ..

[46] Sun F., Pei H.-Y., Hu W.-R., Li X.-Q., Ma C.-X., Pei R.-T. (2013). The cell damage of *Microcystis aeruginosa* in PACl coagulation and floc storage processes. Sep. Purif. Technol..

[47] Sun F., Hu W., Pei H., Li X., Xu X., Ma C. (2015). Evaluation on the dewatering process of cyanobacteria-containing AlCl3 and PACl drinking water sludge. Sep. Purif. Technol..

[48] Li X., Pei H., Hu W., Meng P., Sun F., Ma G., Xu X., Li Y. (2015). The fate of *Microcystis aeruginosa* cells during the ferric chloride coagulation and flocs storage processes. Environ. Technol..

[49] Xu H., Pei H., Xiao H., Jin Y., Li X., Hu W., Ma C., Sun J., Li H. (2016). Behaviors of Microcystis aeruginosa cells during floc storage in drinking water treatment process. Sci. Rep..

[50] Ma C., Pei H., Hu W., Xu H., Jin Y. (2016). The lysis and regrowth of toxic cyanobacteria during storage of achitosan–aluminium chloride composite coagulated sludge: Implications for drinking water sludge treatment. RSC Adv..

[51] Xu H., Pei H., Jin Y., Xiao H., Ma C., Sun J., Li H. (2017). Characteristics of water obtained by dewatering cyanobacteria-containing sludge formed during drinking water treatment, including C-, N-disinfection byproduct formation. Water Res..

[52] Li H., Pei H., Xu H., Jin Y., Sun J. (2018). Behavior of Cylindrospermopsis raciborskii during coagulation and sludge storage-higher potential risk of toxin release than Microcystis aeruginosa?. J. Hazard. Mater..

[53] Water Research Foundation (WRF), Water Research Australia (2015). Optimizing Conventional Treatment for the Removal of Cyanobacteria and Toxins.

[54] Pestana C.J., Reeve P.J., Sawade E., Voldoire C.F., Newton K., Praptiwi R., Collingnon L., Dreyfus J., Hobson P., Gaget V. (2016). Fate of cyanobacteria in drinking water treatment plant lagoon supernatant and sludge. Sci. Total Environ..

[55] Dreyfus J., Monrolin Y., Pestana C.J., Reeve P.J., Sawade E., Newton K., Ho L., Chow C.W.K., Newcombe G. (2016). Identification and assessment of water quality risks associated with sludge supernatant recycling in the presence of cyanobacteria. J. Water Supply Res. Technol.-Aqua.

[56] Jalili F., Trigui H., Guerra Maldonado J.F., Dorner S., Zamyadi A., Shapiro B.J., Terrat Y., Fortin N., Sauvé S., Prévost M. (2021). Can cyanobacterial diversity in the source predict the diversity in sludge and the risk of toxin release in a drinking water treatment plant?. Toxins.

[57] Zamyadi A., Henderson R.K., Stuetz R., Newcombe G., Newtown K., Gladman B. (2016). Cyanobacterial management in full-scale water treatment and recycling processes: Reactive dosing following intensive monitoring. Environ. Sci. Water Res. Technol..

[58] Jalili F. (2022). Optimal Treatment Strategies to Prevent and Manage Cyanobacteria and Cyanotoxins in Drinking Water Sludge. Ph.D. Thesis.

[59] Jalili F., Trigui H., Maldonado J.F.G., Dorner S., Zamyadi A., Shapiro B.J., Terrat Y., Fortin N., Sauvé S., Prévost M. (2022). Oxidation to Control Cyanobacteria and Cyanotoxins in Drinking Water Treatment Plants: Challenges at the Laboratory and Full-Scale Plants. Water.

[60] Zamyadi A., Romanis C., Mills T., Neilan B., Choo F., Coral L.A., Gale D., Newcombe G., Crosbie N., Stuetz R. (2019). Diagnosing water treatment critical control points for cyanobacterial removal: Exploring benefits of combined microscopy, next-generation sequencing, and cell integrity methods. Water Res..

[61] Water Research Foundation (WRF), United States Environmental Protection Agency (US EPA), Veolia Water Indianapolis (2009). Strategies for Controlling and Mitigating Algal Growth within Water Treatment Plants.

[62] Ho L., Barbero A., Dreyfus J., Dixon D.R., Qian F., Scales P.J., Newcombe G. (2013). Behaviour of cyanobacterial bloom material following coagulation and/or sedimentation. J. Water Supply Res. Technol.-Aqua.

[63] Pinkanjananavee K., Teh S.J., Kurobe T., Lam C.H., Tran F., Young T.M. (2021). Potential Impacts on Treated Water Quality of Recycling Dewatered Sludge Supernatant during Harmful Cyanobacterial Blooms. Toxins.

[64] Coral L.A., Zamyadi A., Barbeau B., Bassetti F.J., Lapolli F.R., Prévost M. (2013). Oxidation of Microcystis aeruginosa and Anabaena flos-aquae by ozone: Impacts on cell integrity and chlorination by-product formation. Water Res..

[65] Zamyadi A., Coral L.A., Barbeau B., Dorner S., Lapolli F.R., Prévost M. (2015). Fate of toxic cyanobacterial genera from natural bloom events during ozonation. Water Res..

[66] Ding J., Shi H., Timmons T., Adams C. (2010). Release and removal of microcystins from microcystis during oxidative-, physical-, and UV-based disinfection. J. Environ. Eng..

[67] Zamyadi A., Ho L., Newcombe G., Daly R.I., Burch M., Baker P., Prévost M. (2010). Release and oxidation of cell-bound saxitoxins during chlorination of *Anabaena circinalis* cells. Environ. Sci. Technol..

[68] Fan J.J., Hobson P., Ho L., Daly R., Brookes J. (2014). The effects of various control and water treatment processes on the membrane integrity and toxin fate of *cyanobacteria*. J. Hazard. Mater..

[69] Zhang H., Dan Y., Adams C.D., Shi H., Ma Y., Eichholz T. (2017). Effect of oxidant demand on the release and degradation of microcystin-LR from Microcystis aeruginosa during oxidation. Chemosphere.

[70] Qi J., Lan H., Liu R., Miao S., Liu H., Qu J. (2016). Prechlorination of algae-laden water: The effects of transportation time on cell integrity, algal organic matter release, and chlorinated disinfection byproduct formation. Water Res..

[71] Li X., Chen S., Zeng J., Song W., Yu X. (2020). Comparing the effects of chlorination on membrane integrity and toxin fate of high-and low-viability cyanobacteria. Water Res..

[72] Li X., Chen S., Zeng J., Chabi K., Song W., Xian X., Yu X. (2020). Impact of chlorination on cell inactivation, toxin release and degradation of cyanobacteria of development and maintenance stage. Chem. Eng. J..

[73] Fan J., Rao L., Chiu Y.T., Lin T.F. (2016). Impact of chlorine on the cell integrity and toxin release and degradation of colonial Microcystis. Water Res..

[74] He X., Wert E.C. (2016). Colonial cell disaggregation and intracellular microcystin release following chlorination of naturally occurring Microcystis. Water Res..

[75] Greenstein K.E., Zamyadi A., Glover C.M., Adams C., Rosenfeldt E., Wert E.C. (2020). Delayed Release of Intracellular Microcystin Following Partial Oxidation of Cultured and Naturally Occurring Cyanobacteria. Toxins.

[76] Moradinejad S. (2021). Characterization of Phenomena Governing Oxidation of Cyanobacteria through Metagenomics. Ph.D Thesis.

[77] Zamyadi A., Dorner S., Ndong M., Ellis D., Bolduc A., Bastien C., Prévost M. Breakthrough and accumulation of toxic *cyanobacteria* in full scale clarification and filtration processes. Proceedings of the American Water Works Association-Water Quality Technology Conference and Exposition (WQTC).

[78] Wert E.C., Dong M.M., Rosario-Ortiz F.L. (2013). Using digital flow cytometry to assess the degradation of three cyanobacteria species after oxidation processes. Water Res..

[79] Moradinejad S., Glover C., Mailly J., Zadfatollah-Seighalani T., Peldszus S., Barbeau B., Dorner S., Prévost M., Zamyadi A. (2019). Using advanced spectroscopy and organic matter characterization to evaluate the impact of oxidation on cyanobacteria. Toxins.

[80] Moradinejad S., Glover C., Dorner S., Barbeau B., Prévost M., Zamyadi A. Using advanced spectroscopy and visualization techniques to assess the impact of oxidation on cyanobacteria cell morphology. Proceedings of the WQTC.

[81] Piezer K., Li L., Jeon Y., Kadudula A., Seo Y. (2020). The Application of Potassium Permanganate to Treat Cyanobacteria-Laden Water. Process Saf. Environ. Prot..

[82] Zhou S., Shao Y., Gao N., Deng Y., Qiao J., Ou H., Deng J. (2013). Effects of different algaecides on the photosynthetic capacity, cell integrity and microcystin-LR release of *Microcystis aeruginosa*. Sci. Total Environ..

[83] Xu H., Brookes J., Hobson P., Pei H. (2019). Impact of copper sulphate, potassium permanganate, and hydrogen peroxide on Pseudanabaena galeata cell integrity, release and degradation of 2-methylisoborneol. Water Res..

[84] Zhou T., Cao H., Zheng J., Teng F., Wang X., Lou K., Zhang X., Tao Y. (2020). Suppression of water-bloom cyanobacterium Microcystis aeruginosa by algaecide hydrogen peroxide maximized through programmed cell death. J. Hazard. Mater..

[85] Daly R.I., Ho L., Brookes J.D. (2007). Effect of chlorination on *Microcystis aeruginosa* cell integrity and subsequent microcystin release and degradation. Environ. Sci. Technol..

[86] Matthijs H.C.P., Visser P.M., Reeze B., Meeuse J., Slot P.C., Wijn G., Talens R., Huisman J. (2012). Selective suppression of harmful cyanobacteria in an entire lake with hydrogen peroxide. Water Res..

[87] Foo S.C., Chapman I.J., Hartnell D.M., Turner A.D., Franklin D.J. (2020). Effects of H_2_O_2_ on growth, metabolic activity and membrane integrity in three strains of Microcystis aeruginosa. Environ. Sci. Pollut. Res..

[88] Luukkonen T., Teeriniemi J., Prokkola H., Rämö J., Lassi U. (2014). Chemical aspects of peracetic acid based wastewater disinfection. Water Sa.

[89] Almuhtaram H., Hofmann R. (2022). Evaluation of ultraviolet/peracetic acid to degrade M. aeruginosa and microcystins-LR and-RR. J. Hazard. Mater..

[90] Wang H.-Q., Mao T.-G., Xi B.-D., Zhang L.-Y., Zhou Q.-H. (2015). KMnO4 pre-oxidation for *Microcystis aeruginosa* removal by a low dosage of flocculant. Ecol. Eng..

[91] Barešová M., Načeradská J., Novotná K., Čermáková L., Pivokonský M. (2020). The impact of preozonation on the coagulation of cellular organic matter produced by Microcystis aeruginosa and its toxin degradation. J. Environ. Sci..

[92] Kormas K.A., Lymperopoulou D.S. (2013). Cyanobacterial toxin degrading bacteria: Who are they?. Biomed. Res. Int..

[93] Min H., Golden S.S. (2000). A new circadian class 2 gene, opcA, whose product is important for reductant production at night in Synechococcus elongatus PCC 7942. J. Bacteriol..

[94] Sun J., Xu H., Pei H., Jin Y., Li H., Ma C. (2018). Worse than cell lysis: The resilience of Oscillatoria sp. during sludge storage in drinking water treatment. Water Res..

[95] Pimentel J.S., Giani A. (2014). Microcystin production and regulation under nutrient stress conditions in toxic microcystis strains. Appl. Environ. Microbiol..

[96] Schatz D., Keren Y., Vardi A., Sukenik A., Carmeli S., Börner T., Dittmann E., Kaplan A. (2007). Towards clarification of the biological role of microcystins, a family of cyanobacterial toxins. Environ. Microbiol..

[97] Chow C.W.K., Drikas M., House J., Burch M.D., Velzeboer R.M.A. (1999). The impact of conventional water treatment processes on cells of the cyanobacterium *Microcystis aeruginosa*. Water Res..

[98] United States Environmental Protection Agency (USEPA) (2011). Drinking Water Treatment Plant Residuals Management Technical Report, Summary of Residuals Generation, Treatment, and Disposal at Large Community Water Systems.

[99] Turner T., Wheeler R., Stone A., Oliver I. (2019). Potential Alternative Reuse Pathways for Water Treatment Residuals: Remaining Barriers and Questions—A Review. Water Air Soil Pollut..

[100] Qrenawi L.I., Rabah F.K.J. (2021). Sludge management in water treatment plants: Literature review. Int. J. Environ. Waste Manag..

[101] Ahmad T., Ahmad K., Alam M. (2016). Characterization of Water Treatment Plant’s Sludge and its Safe Disposal Options. Procedia Environ. Sci..

[102] Jung K.W., Hwang M.J., Park D.S., Ahn K.H. (2016). Comprehensive reuse of drinking water treatment residuals in coagulation and adsorption processes. J. Environ. Manag..

[103] Xu Y., Chen T., Xu R., He L., Cui F. (2015). Impact of recycling alum sludge on coagulation of low-turbidity source waters. Desalination Water Treat..

[104] Suman A., Ahmad T., Ahmad K. (2017). Dairy wastewater treatment using water treatment sludge as coagulant: A novel treatment approach. Environ. Dev. Sustain..

[105] Chen W., Song L., Gan N., Li L. (2006). Sorption, degradation and mobility of microcystins in Chinese agriculture soils: Risk assessment for groundwater protection. Environ. Pollut..

[106] Clemente A., Wilson A., Oliveira S., Menezes I., Gois A., Capelo-Neto J. (2020). The role of hydraulic conditions of coagulation and flocculation on the damage of cyanobacteria. Sci. Total Environ..

[107] Pestana C.J., Capelo-Neto J., Lawton L., Oliveira S., Carloto I., Linhares H.P. (2019). The effect of water treatment unit processes on cyanobacterial trichome integrity. Sci. Total Environ..

[108] Pei H., Xu H., Wang J., Jin Y., Xiao H., Ma C., Sun J., Li H. (2017). 16S rRNA Gene amplicon sequencing reveals significant changes in microbial compositions during cyanobacteria-laden drinking water sludge storage. Environ. Sci. Technol..

[109] Chorus I., Welker M. (2021). Toxic Cyanobacteria in Water: A Guide to Their Public Health Consequences, Monitoring and Management.

[110] Chorus I., Bartram J. (1999). Toxic Cyanobacteria in Water: A Guide to Their Public Health Consequences, Monitoring, and Management.

[111] Ellis D. (2009). Guide D’intervention Pour les Propriétaires, les Exploitants ou les Concepteurs de Stations de Production D’eau Potablemunicipales aux Prises avec une Problématique de Fleurs D’eau de Cyanobactéries.

[112] America Water Works Association (AWWA) (2010). Algae Source to Treatment. Manual of Water Supply Practices—M57.

[113] Hawkins P.R., Holliday J., Kathuria A., Bowling L. (2005). Change in cyanobacterial biovolume due to preservation by Lugol’s Iodine. Harmful Algae.

[114] Park J., Kim Y., Kim M., Lee W.H., Park J., Kim Y., Kim M., Lee W.H. (2018). A novel method for cell counting of *Microcystis* colonies in water resources using a digital imaging flow cytometer and microscope. Environ. Eng. Res..

[115] Zamyadi A., McQuaid N., Prévost M., Dorner S. (2012). Monitoring of potentially toxic *cyanobacteria* using an online multi-probe in drinking water sources. J. Environ. Monit..

[116] McQuaid N., Zamyadi A., Prévost M., Bird D.F., Dorner S. (2011). Use of in vivo phycocyanin fluorescence to monitor potential microcystin-producing cyanobacterial biovolume in a drinking water source. J. Environ. Monit..

[117] Gregor J., Marsalek B., Sipkova H. (2007). Detection and estimation of potentially toxic cyanobacteria in raw water at the drinking water treatment plant by in vivo fluorescence method. Water Res..

[118] Zamyadi A., Choo F., Newcombe G., Stuetz R., Henderson R.K. (2016). A review of monitoring technologies for real-time management of cyanobacteria: Recent advances and future direction. TrAC Trends Anal. Chem..

[119] Bowling L.C., Zamyadi A., Henderson R.K. (2016). Assessment of in situ fluorometry to measure cyanobacterial presence in water bodies with diverse cyanobacterial populations. Water Res..

[120] Thomson-Laing G., Puddick J., Wood S.A. (2020). Predicting cyanobacterial biovolumes from phycocyanin fluorescence using a handheld fluorometer in the field. Harmful Algae.

[121] Cotterill V., Hamilton D.P., Puddick J., Suren A., Wood S.A. (2019). Phycocyanin sensors as an early warning system for cyanobacteria blooms concentrations: A case study in the Rotorua lakes. N. Z. J. Mar. Freshw. Res..

[122] World Health Organization (WHO) (1998). Cyanobacterial Toxins: Microcystin-LR in Drinking-Water (Background Document for Development of WHO Guidelines for Drinking Water Quality).

[123] WHO (2020). Cyanobacterial Toxins: Microcystins. Background Document for Development of WHO Guidelines for Drinking-Water Quality and Guidelines for Safe Recreational Water Environments.

[124] Health Canada, Federal-Provincial-Territorial Commitee on Drinking Water (2016). Cyanobacterial toxins in drinking water. Document for public consultation. Guidelines for Canadian Drinking Water Quality: Guideline Technical Document.

[125] Ridal J., Brownlee B., McKenna G., Levac N. (2001). Removal of taste and odour compounds by conventional granular activated carbon filtration. Water Qual. Res. J. Can..

[126] Smith K.M. (2011). Characterization of Activated Carbon for Taste and Odour Control. Master’s Thesis.

[127] Hobson P., Fazekas C., House J., Daly R.I., Kildea T., Giglio S., Burch M., Lin T.-F., Chen Y.-M. (2010). Tastes and Odours in Reservoirs.

[128] Kim K.T., Park Y.-G. (2021). Geosmin and 2-MIB Removal by Full-Scale Drinking Water Treatment Processes in the Republic of Korea. Water.

[129] Zamyadi A., Henderson R., Stuetz R., Hofmann R., Ho L., Newcombe G. (2015). Fate of geosmin and 2-methylisoborneol in full-scale water treatment plants. Water Res..

[130] Sanseverino I., Conduto António D., Loos R., Lettieri T. (2017). Cyanotoxins: Methods and Approaches for Their Analysis and Detection.

[131] Picardo M., Filatova D., Nuñez O., Farré M. (2019). Recent advances in the detection of natural toxins in freshwater environments. TrAC Trends Anal. Chem..

[132] United States Environmental Protection Agency (USEPA) (2016). Method 546: Determination of Total Microcystins and Nodularins in Drinking Water and Ambient Water by Adda Enzyme-Linked Immunosorbent Assay.

[133] Young W.F., Horth H., Crane R., Ogden T., Arnott M. (1996). Taste and odour threshold concentrations of potential potable water contaminants. Water Res..

[134] Watson S.B. (2004). Aquatic taste and odor: A primary signal of drinking-water integrity. J. Toxicol. Environ. Health Part A.

[135] Wert E.C., Korak J.A., Trenholm R.A., Rosario-Ortiz F.L. (2014). Effect of oxidant exposure on the release of intracellular microcystin, MIB, and geosmin from three cyanobacteria species. Water Res..

[136] Chong S., Lee H., An K.-G. (2018). Predicting Taste and Odor Compounds in a Shallow Reservoir Using a Three–Dimensional Hydrodynamic Ecological Model. Water.

[137] Bernhardt H., Clasen J. (1993). Eutrophication control as an essential condition for an optimum disinfection. Water Supply.

[138] Agence Française de Sécurité Sanitaire des Aliments (AFSSA), Agence Française de Sécurité Sanitaire de l’Environnement et du Travail (AFSSET) (2006). Avis relatif à l’évaluation des risques sanitaires liés à la présence de cyanobactéries dans les plans et cours d’eau destinés à la baignade et/ou à d’autres usages.

[139] Pietsch J., Bornmann K., Schmidt W. (2002). Relevance of intra- and extracellular cyanotoxins for drinking water treatment. Acta Hydrochim. Hydrobiol..

[140] Newcombe G., Brooke S., Cullum P., Nicholson B., Slyman N. Oxidation, adsorption and biological treatment for algal toxin removal. Proceedings of the American Water Works Association-Water Quality Technology Conference Seattle.

[141] Newcombe G. (2002). Removal of Algal Toxins from Drinking Water Using Ozone and GAC.

[142] Newcombe G., Drikas M. MIB removal: Adsorption capacity and kinetics of eight activated carbons. Proceedings of the Australian Water and Wastewater Association WaterTECH Conference.

[143] United States Environmental Protection Agency (USEPA) (2019). Cyanobacteria and Cyanotoxins: Information for Drinking Water Systems.

[144] Lusty M.W., Gobler C.J. (2020). The efficacy of hydrogen peroxide in mitigating cyanobacterial blooms and altering microbial communities across four lakes in NY, USA. Toxins.

